# Scope, quality, and inclusivity of clinical guidelines produced early in the covid-19 pandemic: rapid review

**DOI:** 10.1136/bmj.m1936

**Published:** 2020-05-26

**Authors:** Andrew Dagens, Louise Sigfrid, Erhui Cai, Sam Lipworth, Vincent Cheng, Eli Harris, Peter Bannister, Ishmeala Rigby, Peter Horby

**Affiliations:** 1 Epidemic Research Group, University of Oxford, Oxford OX3 7LG, UK; 2 Modernising Medical Microbiology, University of Oxford, Oxford, UK; 3 Centre for Research Synthesis and Decision Analysis, University of Bristol, Bristol, UK; 4 Bodleian Library, University of Oxford, Oxford, UK; 5 School of Medicine, Brighton & Sussex Medical School, Brighton, UK

## Abstract

**Objective:**

To appraise the availability, quality, and inclusivity of clinical guidelines produced in the early stage of the coronavirus disease 2019 (covid-19) pandemic.

**Design:**

Rapid review.

**Data sources:**

Ovid Medline, Ovid Embase, Ovid Global Health, Scopus, Web of Science Core Collection, and WHO Global Index Medicus, searched from inception to 14 Mar 2020. Search strategies applied the CADTH database guidelines search filter, with no limits applied to search results. Further studies were identified through searches of grey literature using the ISARIC network.

**Inclusion criteria:**

Clinical guidelines for the management of covid-19, Middle East respiratory syndrome (MERS), and severe acute respiratory syndrome (SARS) produced by international and national scientific organisations and government and non-governmental organisations relating to global health were included, with no exclusions for language. Regional/hospital guidelines were excluded. Only the earliest version of any guideline was included.

**Quality assessment:**

Quality was assessed using the Appraisal of Guidelines for Research and Evaluation (AGREE) II tool. The quality and contents of early covid-19 guidelines were also compared with recent clinical guidelines for MERS and SARS.

**Results:**

2836 studies were identified, of which 2794 were excluded after screening. Forty two guidelines were considered eligible for inclusion, with 18 being specific to covid-19. Overall, the clinical guidelines lacked detail and covered a narrow range of topics. Recommendations varied in relation to, for example, the use of antiviral drugs. The overall quality was poor, particularly in the domains of stakeholder involvement, applicability, and editorial independence. Links between evidence and recommendations were limited. Minimal provision was made for vulnerable groups such as pregnant women, children, and older people.

**Conclusions:**

Guidelines available early in the covid-19 pandemic had methodological weaknesses and neglected vulnerable groups such as older people. A framework for development of clinical guidelines during public health emergencies is needed to ensure rigorous methods and the inclusion of vulnerable populations.

**Systematic review registration:**

PROSPERO CRD42020167361.

## Introduction

In late 2019 a novel coronavirus, severe acute respiratory syndrome coronavirus 2 (SARS-CoV-2), causing an acute respiratory disease, coronavirus disease 2019 (covid-19), spread from its origins in China to become a pandemic. As of 26 March 2020, 455 770 cases had been identified worldwide, causing 20 740 deaths. No successful therapeutic intervention for covid-19 has yet been established, so supportive care is the most important aspect of clinical management, supporting the patient’s physiology to aid recovery. Optimal provision of supportive care is therefore fundamental both to the wellbeing of individual patients and to securing the confidence of the general population. To enable the provision of best care, clinicians need evidence based recommendations developed using accepted methods. Such clinical guidelines must be readily available, of good quality, and inclusive of vulnerable patient groups.

Clinical guidelines are defined as “systematically developed statements to assist practitioner and patient decisions about appropriate healthcare for specific clinical circumstances.”^1^ Widely agreed, rigorous methods now exist for the production and appraisal of clinical guidelines. The Appraisal of Guidelines for Research and Evaluation (AGREE) II tool is the most widely used guideline appraisal tool,^2^ and it has become the international “gold standard” for guideline development.

During times of crisis, guidelines from the World Health Organization may be the only source of direction available to clinicians globally. They may be adopted internationally with only minor local adaptations. Thus, WHO guidelines must be of the highest possible standard. However, inherent uncertainty exists in the early phase of a pandemic, which, when combined with the considerable pressure to act rapidly, makes the production of gold standard guidelines very challenging. That studies have repeatedly shown WHO guidelines produced in emergencies and in non-emergencies to score poorly in objective appraisals of their methods is therefore not surprising.^3^ ^4^ Often, they do not adhere even to WHO’s internal standard procedures.

Inclusivity is also vital in a pandemic; covid-19 manifests differently in different patient groups, being most severe among older people and those with comorbidities.^5^ Furthermore, the pandemic has now moved to low resource settings, where logistical challenges to a public health emergency are greater. Accordingly, clinical guidelines need to be inclusive of different groups and different resource settings.

This rapid review aimed to assess the availability, quality, and inclusivity of clinical guidelines produced early in the covid-19 pandemic. To our knowledge, this is the first review of clinical management guidelines produced during a pandemic.

## Methods

This study was a rapid review of clinical guidelines for the management of covid-19 produced early in the pandemic. We defined clinical guidelines as systematically developed recommendations produced to direct the management of patients with confirmed or suspected covid-19. To be included, guidelines had to make specific recommendations aimed at the clinical care of patients—for example, concerning fluid resuscitation, oxygen provision, or a therapeutic intervention. We excluded guidelines that exclusively concerned prevention and control of infection or diagnostic studies.

The study was nested within an extensive systematic review of supportive care in high consequence infectious diseases. That larger study is registered with the PROSPERO international prospective register of systematic reviews (CRD42020167361) and follows Preferred Reporting Items for Systematic Reviews and Meta-Analyses (PRISMA) guidelines on the conduct of systematic reviews (supplementary material). In light of the global covid-19 pandemic, we opted to produce a nested rapid review of guidelines on covid-19 by using a modified protocol for rapid reviews.^6^

We included guidelines produced by international and national scientific organisations and government and non-governmental organisations relating to global health. We made no exclusions for language. We excluded regional/hospital guidelines to make the search feasible. We included only the earliest version of any guideline.

We searched the following databases from inception to search date (14 February 2020) for relevant studies: Ovid Medline, Ovid Embase, Ovid Global Health, Scopus, Web of Science Core Collection, and WHO Global Index Medicus. The search strategies applied the CADTH database guidelines search filter to text words and relevant index terms.^7^ We applied no limits to the search results. The full search strategies are shown in the supplementary material. We identified further studies through searches constructed using Google Scholar and the PROSPERO database of registered systematic reviews. We augmented this with an extensive grey literature search that continued until 14 March 2020. We requested guidelines from the Ministry of Health of each G20 nation where none was available on their respective websites. We also used the International Severe Acute Respiratory and Emerging Infections Consortium (ISARIC), an international clinical research network for infectious disease.

To facilitate a more rapid review, one reviewer independently screened the title and abstract of all references. A second reviewer screened 10% of excluded references for quality control. After each reference passed the first screening stage, two reviewers screened the full text independently. Where conflict about inclusion existed, a third reviewer made the final decision.

We extracted data by using the methodological guide produced by Johnston et al.^8^ The team members speak multiple languages; as a last resort when no fluent speaker was available we used Google Translate. We used a standardised form for data extraction. We used Distiller SR (Evidence Partners, Ottawa, Canada) and Microsoft Excel for all screening and data extraction. For each guideline, we extracted data on source, year of production, clinical topics covered, and the patient demographic.

Two reviewers independently appraised each eligible guideline by using the AGREE II instrument according to the instructions of the AGREE Research Trust.^2^ The AGREE II instrument provides an objective framework to assess the quality of clinical guidelines; it consists of six domains and two global rating items. The six domains are scope and purpose, stakeholder involvement, rigour of development, clarity of presentation, applicability, and editorial independence. Each domain is assessed on the basis of several “items,” of which there are 23 in total. The score is completed by at least two independent assessors on a seven point scale. Total scores are scaled to a percentage of the maximum score in each domain; 100% is achieved if each reviewer scores 7 for all items in a domain. The domain would score 0% if each reviewer scored 1 (the minimum value) for all items in the domain.

### Patient and public involvement

There was no public or patient involvement in the course of this project. However, extensive involvement is planned in the wider systematic review of which this review was a part.

## Results

**Fig 1:**
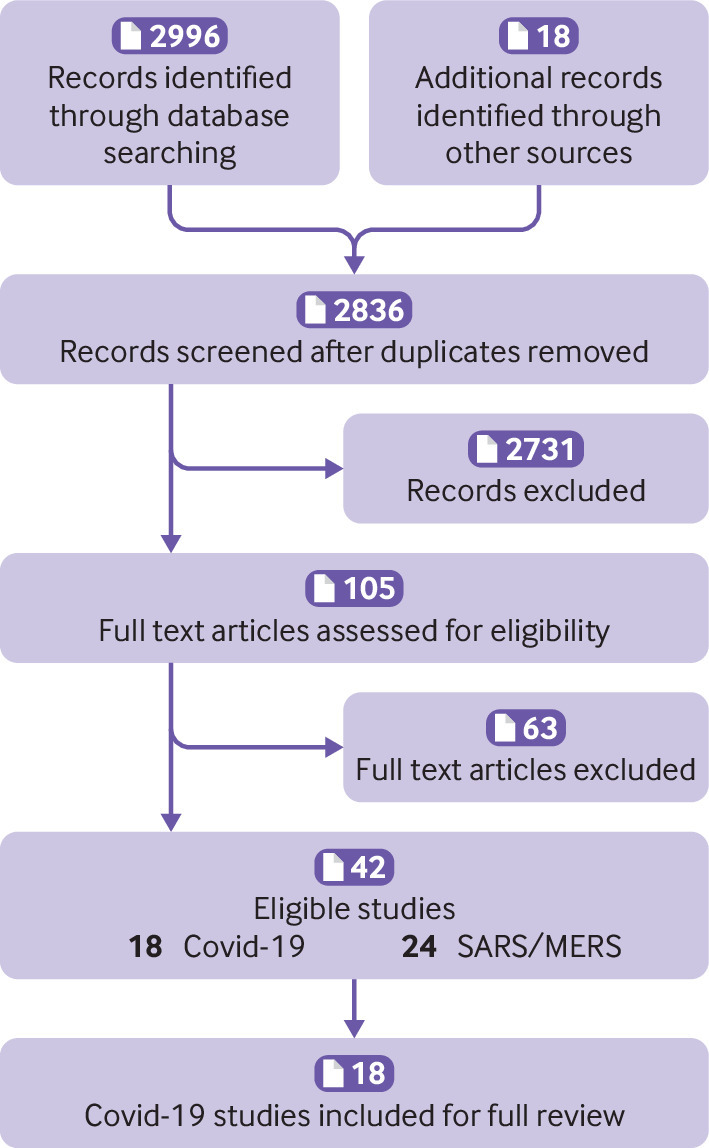
PRISMA diagram. MERS=Middle East respiratory syndrome; SARS=severe acute respiratory syndrome

In total, we identified 2996 records through database searching and a further 18 through grey literature searches. We excluded 2731 (96%) studies after de-duplication and title screening and a further 63 (60%) after further screening of the full text. Forty two guidelines proceeded to data extraction and synthesis, of which 18 directly pertained to covid-19 and 24 were guidelines relating to severe acute respiratory syndrome (SARS) or Middle East respiratory syndrome (MERS) by national organisations promoted in the covid-19 response (fig 1).

We identified 18 national guidelines on covid-19, most of which were published in an upper middle income or high income country (table 1).^8^ ^9^ ^10^ ^11^ ^13^ ^14^ ^15^ ^16^ ^18^ ^19^ ^20^ ^21^ ^22^ ^23^ ^24^ ^25^ ^26^ We did not find a guideline produced in a low income country.

**Table 1:** Availability of clinical management guidelines for COVID-19 by resource setting (World Bank Classification)

Guideline	Income group of country			
	Low	Lower middle	Upper middle	High
World Health Organization^9^				
Ministry of Health, Brazil^10^			X	
National Health Commission, China^5^			X	
COREB mission nationale, France^11^				X
Robert Koch Institute, Germany^12^				X
Ministry of Health, Netherlands^13^				X
Ministry of Health and Family Welfare, India^14^		X		
Ministry of Health, Indonesia^13^		X		
Società Italiana di Malattie Infettive e Tropicali, Italy^15^				X
Japanese Association of Infectious Diseases, Japan^16^				X
Department of Public Health, Malaysia^17^			X	
Working group on COVID 2019, Russia^18^			X	
Centre for Disease Control, Saudi Arabia^19^				X
Central COVID Task Force, South Korea^20^				X
Ministry of Health, Spain^21^				X
Center for Disease Control, Taiwan^22^				X
Ministry of Health, Turkey^23^			X	
Centers for Disease Control and Prevention, USA^24^				X

Often clinical guidelines were embedded within a document that primarily focused on infection control. Generally, the clinical recommendations provided by the guidelines were non-specific and covered a narrow range of topics (table 2). It was evident that most countries relied heavily on WHO guidelines in formulating their own guidelines.

**Table 2:** Clinical content of international guidelines produced in early covid-19 pandemic

Guidelines	Basic resuscitation				VTE, DVT prophylaxis	Infection and IPC				Advanced resuscitation				Avoid*		Other
	Fluid resuscitation	Supplemental oxygen	Nutrition	Symptom control, including NSAIDs		IPC	Empirical antibiotics	Antiviral therapy		Intubation and ventilation	RRT	Vasopressors and inotropes		Steroids	NIV	
WHO^9^	X	X			X	X	X			X		X		X	X	
Brazil^10^	X	X			X	X	X			X		X		X		
China^5^	X	X	X			X	X	X		X		X				Traditional Chinese medicine
France^11^						X	X	X		X				X	X	
Germany^12^	X	X				X	X	X						X		
Netherlands^26^†								X								
India^14^	X	X			X	X	X			X		X		X	X	Early prone positioning
Indonesia^13^	X	X			X	X	X					X		X	X	
Italy^15^							X	X						X	X	
Japan^16^						X		X								
Malaysia^17^						X	X							X		
Russia^18^	X	X		X		X	X	X		X						
Saudi Arabia^19^‡						X										
South Korea^20^†								X						X		
Spain^21^	X	X			X	X	X	X		X		X		X	X	Early prone positioning
Taiwan^22^	X	X				X	X			X		X		X	X	
Turkey^23^	X	X				X				X				X		
US CDC^24^‡	X	X				X	X			X		X		X		

CDC=Centers for Disease Control and Prevention; DVT=deep venous thrombosis; IPC=infection prevention and control; NIV=non-invasive ventilation; NSAID=non-steroidal anti-inflammatory drug; RRT=renal replacement therapy; VTE=venous thromboembolism; WHO=World Health Organization.

* Guidelines specifically recommend against particular intervention.

† Provides no original supportive care guidelines; refers user directly to WHO clinical guidelines and Surviving Sepsis.

‡ Focus of guidelines was on antiviral drugs.

The format of the supportive care recommendations in the guidelines varied widely, ranging from brief notes or flow diagrams to lengthy, nuanced descriptions of therapeutic options. Emphasis differed among the guidelines, with some being more conservative than others and with variation in specific recommendations such as the choice of antiviral drugs (table 3). Very few guidelines made specific recommendations on the use of treatments for symptom control such as non-steroidal anti-inflammatory drugs. Recommendations on the use of non-invasive ventilation varied widely (table 4).

**Table 3:** Variability in recommendations of targeted covid-19 therapies across guidelines

Country	Antivirals	Level of support	Notes
Italy	If need for oxygen or clinical worsening: remdesivir ampoules 150 mg 1 day 200 mg IV in 30 min, then 100 mg IV OD for another 9 days in combination with chloroquine 500 mg BD or hydroxychloroquine 200 mg BD (duration of treatment 5-20 days)	Expert consensus following literature review	Methods for reaching conclusions unclear
	In severe disease: remdesivir 1 day 200 mg IV, then 100 mg/day IV (days 2-10) + chloroquine 500 mg BD or hydroxychloroquine 200 mg × 2 PO 5-20 days		
Russia	In moderate to severe infections: 400 mg lopinavir/100 mg ritonavir BD for 14 days PO; or 400 mg lopinavir/100 mg ritonavir) (5 mL) BD 14 days NGT; or recombinant interferon 1b 0.25 mg/mL (8 million IU) SC every second day for 14 days; or ribavarin 2 g loading dose, then 1200 mg TID for 4 days, then 4-6 days 600 mg TID	Results from literature review led to three drugs being chosen. No preference or order is recommended. Not clear how authors excluded other options	Advises antivirals can be prescribed off label after benefits *v* risk assessment. Oseltamivir not recommended
France	Advised for all patients admitted to ICU on confirmation of diagnosis. First line: lopinavir/ritonavir 400 mg BD; second line: hydroxychloroquine 200 mg BD	If parenchymal involvement. Recommendations based on data in SARS and MERS. First line treatment chosen because readily available	Advises against ribavarin owing to inconclusive data
Netherlands	In moderate disease: first line chloroquine 600 mg PO, then 300 mg for 5 days; second line lopinavir/ritonavir 400/100 mg BD for 14 days	Noting that very little information is available, makes no definitive recommendations. Acknowledges lack of phase I data for remdesivir	Advises against use of ribavirin alone owing to toxicity at required doses. Notes poor evidence for interferon in combination with ribavirin. Oseltamivir not recommended
	In severe disease: remdesivir + chloroquine or lopinavir/ritonavir + chloroquine		
Spain	First line: lopinavir/ritonavir 400/100 mg BD PO until disappearance of fever for maximum 14 days; second line: interferon β1b 0.25 mg SC every 48 h for 14 days or interferon α2b 5 million units in 2 mL of sterile serum, BD INH	Only for severe pneumonia, CURB >65, SpO_2_ <90%	Notes in-vitro studies and ongoing Chinese trials. Oseltamivir not recommended
	Remdesivir 200 mg IV, then 100 mg IV OD for 9 days	For compassionate use only in severe disease	
China	Alpha-interferon (5 million units or equivalent dose BD INH) or lopinavir/ritonavir (200/50 mg × 2 BD for ≤10 days); or ribavirin (used jointly with interferon or lopinavir/ritonavir, 500 mg IV TID for adults, for ≤10 days); or chloroquine phosphate (500 mg BD for ≤10 days); or arbidol (200 mg TID for adults, for ≤10 days)		Does not recommend using three or more antiviral drugs at same time
Germany	Numerous antiviral therapies are used in the context of SARS-CoV-2. Too little data are currently available to make a therapy recommendation in Germany. Even for severe forms of COVID-19 there is insufficient evidence to recommend therapy		
Japan	No specific therapy recommended. Lopinavir/ritonavir, anti-influenza drug favipiravir, remdesivir, and ciclesonide, an inhaled steroid used in asthma, are listed as potential therapeutic agents		Advises these agents may be future therapeutic agents pending trials
South Korea	Lopinavir/ritonavir 400/100 mg BD for 7-10 days; or hydroxychloroquine 400 mg OD; or interferon can be administered in combination with lopinavir/ritonavir	Remdesivir only to be used in clinical trials	Ribavirin not recommended owing to adverse reactions

BD=twice daily; ICU=intensive care unit; INH=inhalation; IV=intravenous; MERS=Middle East respiratory syndrome; NGT=nasogastric tube; OD=once daily; PO=oral; SARS-CoV-2=severe acute respiratory disorder coronavirus 2; SC=subcutaneous; TID=three times daily;

**Table 4:** Recommendations on use of high flow nasal oxygen (HFNO) and non-invasive ventilation (NIV) in covid-19 from clinical guidelines available early in pandemic

Guideline	Recommendations
World Health Organization^9^	High flow nasal oxygen and non-invasive ventilation should be used only in selected patients with hypoxaemic respiratory failure
	Limited data suggest a high failure rate in patients with other viral infections such as MERS-CoV who receive NIV
	Patients receiving a trial of NIV should be in a monitored setting and cared for by experienced personnel capable of endotracheal intubation in case the patient acutely deteriorates or does not improve after a short trial (about 1 hour). Patients with haemodynamic instability, multi-organ failure, or abnormal mental status should likely not receive NIV in place of other options such as invasive ventilation
	Owing to uncertainty around the potential for aerosolisation, high flow oxygen and NIV, including bubble CPAP, should be used with airborne precautions until further evaluation of the safety can be completed
Ministry of Health, Brazil^10^	Consider NIV if mild respiratory distress
	Proceed with endotracheal intubation if there is no response to NIV using aerosol precautions
National Health Commission, China^25^	Timely provision of effective oxygen therapy, including nasal catheter and mask oxygenation, and if necessary, nasal high flow oxygen therapy
	When respiratory distress and/or hypoxaemia of the patient cannot be alleviated after receipt of standard oxygen therapy, high flow nasal cannula oxygen therapy or NIV can be considered. If conditions do not improve or even get worse within a short time (1-2 hours), tracheal intubation and invasive mechanical ventilation should be used in a timely manner
COREB mission nationale, France^11^	In general, techniques at risk of aerosolisation risk contamination of personnel and must be avoided as much as possible (NIV, HFNO)
	In situations where NIV is still necessary, care givers must wear PPE and the patient must wear a mask. The NIV must be stopped before the mask is removed from the patient. Limit the presence of care givers in the rooms of infected patients receiving treatment with NIV or optiflow (HFNO)
Robert Koch Institute, Germany^12^	Early administration of oxygen, possibly non-invasive or invasive ventilation
	It is important to acknowledge that oxygen supplementation through high flow nasal cannula (HFNC) and NIV leads to aerosol formation. It is therefore absolutely necessary to make sure that HFNC and facemasks are fitted correctly to the patient, and that the medical personnel at the bedside strictly adhere to PPE instructions. NIV with a helmet should be preferred where available
	In general, we advise medical professionals to be rather restrictive with HFNC and NIV in the context of covid-19. In patients with severe hypoxemia (PaO_2_/FiO_2_ ≤200 mm Hg) we suggest performing early intubation and invasive mechanical ventilation. In any case, continuous monitoring and preparedness for urgent intubation are cornerstones in the treatment of patients with covid-19 with respiratory failure. A delay in intubation in patients failing NIV worsens outcome, and any emergency intubation in this cohort puts medical professionals at risk and should be avoided
Ministry of Health, Holland^26^	No specific guidance
Ministry of Health and Family Welfare, India^14^	The risk of treatment failure is high in patients with MERS treated with NIV, and patients treated with either HFNO or NIV should be closely monitored for clinical deterioration
	Recent publications suggest that newer HFNO and NIV systems with good interface fitting do not create widespread dispersion of exhaled air and therefore should be associated with low risk of airborne transmission
Ministry of Health, Indonesia^13^	The use of NIV is not recommended in pandemic viral disease, because this causes delays in intubation, large tidal volume, and parenchymal injury. The available data, although limited, show the level of failure is high when MERS patients have oxygen therapy with NIV
	Recent publications show that HFNO and NIV systems use an interface that matches the face so the risk of airborne transmission when patient expires is low
Società Italiana di Malattie Infettive e Tropicali, Italy^15^	There is strong evidence that the use of NIV in the treatment of covid-19 pneumonia is associated with a worse outcome. On this basis, WHO recommends, where possible, avoidance of NIV and adoption instead of standards that provide for early intubation. If NIV is used, this must be done within an intensive care unit
Japanese Association of Infectious Diseases, Japan^16^	No specific guidance
Department of Public Health, Malaysia^17^	No specific guidance
Working group on COVID 2019, Russia^18^	It is permissible to use NIV as the beginning of respiratory support in patients with acute respiratory distress
	With the ineffectiveness of NIV—hypoxaemia, metabolic acidosis or no increase in the PaO_2_/FiO_2_ index in 2 hours, high breathing (desynchronisation with a respirator, participation of auxiliary muscles, “failures” during triggering of inspiration on pressure-time curve)—tracheal intubation is indicated
Centre for Disease Control, Saudi Arabia^19^	No specific guidance; refers to WHO
Central COVID Task Force, South Korea^20^	No specific guidance
Ministry of Health, Spain^21^	HFNO and NIV should be reserved for very specific patients. NIV should under no circumstances delay the indication of intubation. Treatment failure with NIV in MERS was high. Patients with NIV and HFNO should be closely monitored and prepared for possible intubation
Center for Disease Control, Taiwan^22^	Neither HFNO nor NIV is recommended for routine use in SARS-CoV-2 infected patients
	According to the treatment experience of MERS patients, the treatment failure rate using NIV is high
	Risks associated with NIV include delayed intubation, excessive tidal volume, injurious transpulmonary pressure, and haemodynamic instability
Ministry of Health, Turkey^23^	No specific guidance
Centers for Disease Control and Prevention, USA^24^	No specific guidance

CPAP=continuous positive airway pressure; MERS-CoV=Middle East respiratory syndrome coronavirus; PPE=personal protective equipment; SARS-CoV-2=severe acute respiratory syndrome coronavirus 2.

**Fig 2:**
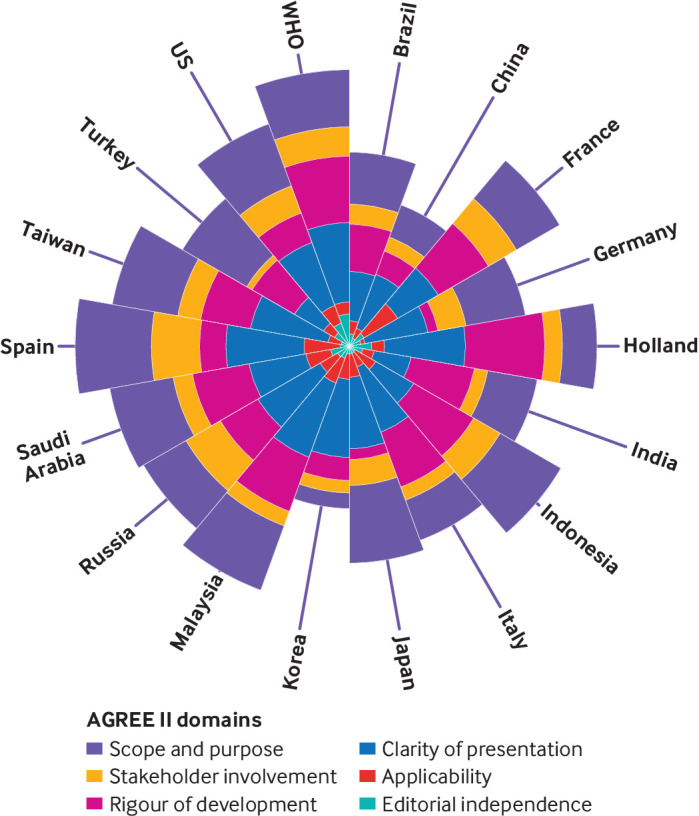
Total Appraisal of Guidelines for Research and Evaluation (AGREE) II scores by domain across 18 national guidelines

Overall quality as assessed by the AGREE II tool was poor (fig 2). The stacked polar chart shows the sum of the total AGREE II scores with sub-bars, representing six domains (100 for each domain), stacked end to end for each country. WHO guidelines were rated as 265.42 (44%) out of 600 in total. Clinical guidelines produced in Spain (260; 43%)) and in Malaysia (248; 41%) scored particularly highly for methodological rigour, whereas the guidelines produced in China (145; 24%) and South Korea (156; 26%) scored particularly poorly. Domains in which all of the guidelines scored poorly were stakeholder involvement, applicability, and editorial independence.

We observed a lack of clear links between the evidence base and recommendations throughout the guidelines globally—for instance, in the strong discouragement of the use of steroids or the use of antimicrobials (table 5). Antimicrobial recommendations also varied, with several guidelines recommending empirical antimicrobial treatment for all patients with severe acute respiratory symptoms and others recommending it only on the basis of clinical aetiology.

**Table 5:** Recommendations for use of corticosteroids for covid-19 in global guidelines produced early in pandemic

Origin	Corticosteroid recommendations	Evidence base	Antimicrobials notes
WHO	Corticosteroid therapy contraindicated	Stockman LJ et al,^27^ Rodrigo C et al,^28^ Delaney et al,^29^ Arabi YM et al^30^	Give empirical antimicrobials to treat all likely pathogens causing SARI
Italy	Not recommended for confirmed covid-19 patients, but low dose dexamethasone may be considered in patients with confirmed ARDS on ICU clinicians’ indication	World Health Organization interim guidance,^9^ Villar J et al^31^	Add antibiotic (empirical or targeted) according to clinical indications, health policies, or protocols in use
US CDC	Corticosteroids should be avoided unless indicated for other reasons (eg, COPD exacerbation or septic shock)	Zumla A et a,l^32^ Arabi YM et al,^30^ Russell et al,^33^ Metlay JP et al^34^	
India	Not recommended for viral pneumonia or ARDS outside of clinical trials, unless indicated for other reason	No link to supporting evidence provided	Antibiotics not recommended/covered
Turkey	Not recommended routinely	No link to supporting evidence provided	Give empirical antimicrobials to treat all likely pathogens causing SARI
South Korea	Steroids not indicated in general but may be considered for other conditions, such as septic shock	No link to supporting evidence provided	Empirical antimicrobials for possible pathogens are recommended
France	Steroids not indicated for SARS-CoV-2 infection alone	Stockman LJ et al^27^	Routine use of antibiotics for treatment of covid-19 not recommended. However, antibiotics may be used if accompanying bacterial infection is suspected
Brazil	Not recommended for viral pneumonia or ARDS outside of clinical trials, unless indicated for other reasons	No link to supporting evidence provided	
Taiwan	Not recommended for viral pneumonia or ARDS outside of clinical trials, unless indicated for other reasons	No link to supporting evidence	Systematic coverage of bacterial infection/superinfection recommended in severe forms
Indonesia	Not recommended for viral pneumonia or ARDS outside of clinical trials, unless indicated for other reasons	No clear link to supporting evidence	
Spain	Not recommended	No clear link to supporting evidence	Give empirical antimicrobials to treat all likely pathogens that cause SARS
Malaysia	Not recommended unless indicated for other reasons (eg, COPD, septic shock)	No clear link to supporting evidence	Consider giving empirical antibiotics to treat other possible bacterial infection
Germany	Not recommended without clear indication	No clear link to supporting evidence	Give empirical antibiotics based on likely aetiology

ARDS=acute respiratory distress syndrome; COPD=chronic obstructive pulmonary disease; ICU=intensive care unit; SARI=severe acute respiratory illness; SARS-CoV-2=severe acute respiratory syndrome coronavirus 2.

Globally, very few recommendations were made on prophylaxis for venous thromboemolism (table 6). Some guidelines linked their recommendations to a consideration of the published literature, but many did not. Even where an explicit link was made, no systematic weighting for that evidence (for example, Grading of Recommendations, Assessment, Delivery and Evaluations (GRADE)) was used.

**Table 6:** Recommendations on use of venous thromboembolism (VTE) prophylaxis

Guideline	VTE prophylaxis recommendations	Notes
World Health Organization^9^	Use pharmacological prophylaxis (low molecular weight heparin (preferred if available) or heparin 5000 units subcutaneously twice daily) in adolescents and adults without contraindications. For those with contraindications, use mechanical prophylaxis (intermittent pneumatic compression devices)	No clear link to supportive evidence. Some indication that recommendations are based on previously published guidelines
Ministry of Health, Brazil^10^	Use pharmacological prophylaxis in patients without contraindications. If there are contraindications, use mechanical prophylaxis	Based on WHO guidelines
National Health Commission, China^25^	No specific advice given	
COREB mission nationale, France^11^	No specific advice given	
Robert Koch Institute, Germany^12^	No specific advice given	
Ministry of Health, Holland^26^	No specific advice given	
Ministry of Health and Family Welfare, India^14^	Use pharmacological prophylaxis (low molecular weight heparin (preferred if available) or heparin 5000 units subcutaneously twice daily) in adolescents and adults without contraindications. For those with contraindications, use mechanical prophylaxis (intermittent pneumatic compression devices)	No clear link to supportive evidence. Some indication that recommendations are based on previously published guidelines
Ministry of Health, Indonesia^13^	Use prophylactic drugs (low molecular weight heparin if available, or 5000 subcutaneous heparin units twice a day) in adolescent and adult patients when no contraindications. If there are contraindications use mechanical prophylaxis	No clear link to supportive evidence
Società Italiana di Malattie Infettive e Tropicali, Italy^15^	No specific advice given	
Japanese Association of Infectious Diseases, Japan^16^	No specific advice given	
Department of Public Health, Malaysia^17^	No specific advice given	
Working group on COVID 2019, Russia^18^	No specific advice given	
Centre for Disease Control, Saudi Arabia^19^	No specific advice given	
Central COVID Task Force, South Korea^20^	No specific advice given	
Ministry of Health, Spain^21^	Efforts will be made to avoid the complications listed—pulmonary thromboembolism: prophylactic anticoagulation	No clear link to supportive evidence
Center for Disease Control, Taiwan^22^	No specific advice given	
Ministry of Health, Turkey^23^	No specific advice given	
Centers for Disease Control and Prevention, USA^24^	No specific advice given	

**Fig 3:**
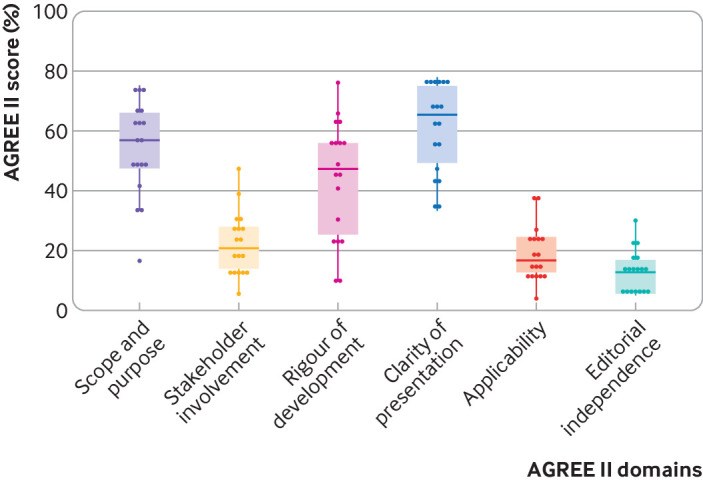
Combined Appraisal of Guidelines for Research and Evaluation (AGREE) II assessment for all guidelines (n=18) as percentages of maximum possible score per domain. Vertical lines indicate range; horizontal line represents mean score for each domain

We found wide variations across individual score domains when comparing guidelines. None of the guidelines scored above 50% for the domains on editorial independence, applicability, or stakeholder involvement (fig 3). The score for rigour of development, a key component for evidence based guidelines, ranged from 10% to 76%. We could find no examples of a systematic review being done, most guidelines did not grade the strength of their recommendations, and little description existed of how these recommendations were made. We found no evidence of a guideline being externally reviewed before release. The guidelines made little provision for vulnerable groups such as pregnant women and children, and few recommendations pertained to the care of older people and immunocompromised patients (table 7).^9^ ^10^ ^11^ ^12^ ^13^ ^15^ ^16^ ^17^ ^18^ ^19^ ^20^ ^21^ ^22^ ^23^ ^24^ ^25^ ^26^

**Table 7:** Vulnerable groups covered by clinical guidelines available early in covid-19 pandemic

Origin	Children	Pregnant women	HIV/immunocompromised	Older people	Adults
WHO^9^	X	X			X
Brazil^10^	X	X			X
China^25^					X
France^11^					X
Germany^12^		X			X
Netherlands^26^	X	X			X
India^19^	X	X	X		X
Indonesia^13^	X		X	X	X
Italy^15^					X
Japan^16^					X
Malaysia^17^	X	X			X
Russia^18^	X	X			X
Saudi Arabia^19^					X
South Korea^20^					X
Spain^21^	X				X
Taiwan^22^	X	X			X
Turkey^23^					X
United States^24^		X			X

We compared the quality and content of WHO guidelines for MERS with the current interim WHO guidelines on covid-19 (table 8). The covid-19 guidelines had significantly lower scores than the MERS guidelines in all AGREE II domains, except the domain of rigour of development. Both guidelines followed similar case definitions.

**Table 8:** Appraisal of Guidelines for Research and Evaluation (AGREE) II scores of World Health Organization covid-19 guidelines produced early in pandemic versus current Middle East respiratory syndrome (MERS) guidelines, as percentage of maximum possible score

Origin	Scope and purpose	Stakeholder involvement	Rigour of development	Clarity of presentation	Applicability	Editorial independence	Overall
WHO covid-19^9^	55	27	64	75	12	30	4
WHO MERS^35^	75	45	36	97	41	33	5

WHO produces a handbook for internal guideline development, including details of how it produces interim guidance.^36^ WHO states that “although the target audience or other stakeholders may demand that interim guidance be generated quickly, this type of guideline fully complies with all processes and procedures and meets the standards set out in this handbook.”

However, our evaluation suggests that the WHO MERS guidelines, originally published in 2013 and now in their third version, continue to fail to score highly in the domains of applicability, editorial independence, and stakeholder involvement. This is echoed in the interim guidance for covid-19, which also scored poorly in these domains. The low scores are caused by little discussion of the applicability of the guidelines, inadequate recording of conflicts of interest, a narrow range of included stakeholders, and insufficient planning for updating the document.

The WHO covid-19 interim guidelines were based on the early MERS guidelines and are very similar in their recommendations. Considerable overlap in recommendations may exist because a betacoronavirus causes both MERS and covid-19, and other guidelines on viral respiratory infections may also have applicable elements. Our search found alternative guidelines for other respiratory infections that may be applicable and of high quality (table 9).

**Table 9:** Possible alternatives to World Health Organization interim guidelines for pandemic acute respiratory infections

Source	Notes
GTEI guidelines on the treatment and management of influenza A/H1N1^37^	High quality and very extensive guidelines on ITU management of patients with influenza
BTS guidelines on the management of pandemic influenza^38^	Extensive and potentially applicable to covid-19
ERS guidelines on the management of adult lower respiratory tract infections^39^	

BTS=British Thoracic Society; ERS=European Respiratory Society; GTEI=infectious diseases working group; ITU=intensive care unit.

## Discussion

As the covid-19 pandemic grows, clinical guidelines will be in increasing demand globally. This rapid review contains lessons for both the current pandemic and future pandemics. We found shortcomings in the international body of clinical guidelines covid-19 produced early in the pandemic. Very few organisations constructed their own guidelines independently, meaning that nearly all guidelines incorporated the WHO interim guidance at least partially. Lack of reporting of the process of obtaining evidence and reaching recommendations made assessment of the appropriateness and quality of the recommendations for individual users and organisations difficult.

Clearly, these guidelines were made under conditions of uncertainty at a time of international crisis. Moreover, elements of AGREE II may be ill suited to the demands of guideline production during the current crisis. Nevertheless, well constructed, evidence based clinical guidelines are crucial to the response to covid-19, to help to guide clinical decision making and improve patients’ outcomes. Clinicians need to be able to rely on the editorial independence of the guidelines they use, but declarations of interest were poorly documented in the early international covid-19 guidelines. In “peacetime,” declaring conflicts of interest is a vital component of both GRADE and WHO-INTEGRATE Evidence to Decision frameworks,^40^ so why not during a pandemic? Full disclosure of conflicts of interest is not time consuming and is important when making recommendations on novel or experimental treatment on the basis of limited or no evidence.

Furthermore, given the complexity of the global health emergency, it seems reasonable that all guideline writers should seek to include as broad a range of stakeholders as possible. Given the resource constraints faced, matters of affordability and availability within health systems should be covered. Finally, almost none of the published guidelines we reviewed reported any mechanism for updates, audit, and monitoring. The covid-19 pandemic is rapidly evolving, and under these circumstances provisions for audit and monitoring of any guideline are crucial.

### Variation in recommendations

The limited level of rigour in constructing the guidelines made accounting for the notable variation in the recommendations difficult. For instance, the Russian guidelines advocated the use of anti-inflammatory drugs whereas most others made no such recommendations.^18^ Most guidelines strongly discouraged the use of steroids for covid-19. However, the detail with which this recommendation was made varied widely. The use of steroids in acute respiratory infections such as covid-19 is contested, but the debate is complex and relies on the interpretation of observational studies and surrogate outcomes.^33^ ^41^ Guidelines must be clear but must not obscure the complexity of this debate to guideline users.

The most marked difference in the content of the guidelines was in the support for antiviral agents, both in terms of whether to use antivirals at all and in the specific antiviral regimen endorsed. Clearly, complex factors are involved in choosing a treatment regimen in an emergency. However, because the guidelines were drafted without clear links between recommendation and underlying evidence, the logic of each regimen was hard to ascertain. Expecting strongly evidenced interventions for a recently emerged disease is unreasonable, and we appreciate that more thorough and a greater number of guidelines will be produced as the pandemic progresses. However, clinicians need guidelines that are evidence based and include a thorough evaluation of the level of evidence on which a recommendation is based, while also conveying which populations and indications the guidance applies to. When no evidence is available, this should also be made clear. Any recommendations made should be directly linked to an evaluation of the supporting evidence. GRADE is a systematic method for making clinical practice recommendations and helps to portray the certainty with which a recommendation is made and could be used.^40^ Arguably, the variation seen in the recommendations on the use of supportive care and the lack of recommendations for vulnerable, high risk populations underline the importance of a gold standard framework for guideline construction under conditions of uncertainty.

All of the covid-19 guidelines found were produced in high income or upper middle income countries, and therefore include assumptions about technology that may not be realistic in low income settings. For instance, avoiding non-invasive ventilation in favour of early intubation and prone positioning might reflect the clinical gold standard in some countries, but it is clearly heavily resource dependent. This must be tackled as the pandemic moves into lower resourced settings.

The early covid-19 guidelines showed a lack of inclusivity. We sought to explore whether the guidelines incorporated the needs of vulnerable groups, defined broadly to include older people, children, pregnant women, and patients with comorbidities. We found that some groups are only cursorily covered in the guidelines or not mentioned at all. Few guidelines explicitly described care for older or immunocompromised people, who represent vulnerable groups with unique needs.

### Limitations of study

This review has some limitations. Firstly, most guidelines were published outside of bibliographic databases. Although we did an extensive search of the grey literature, our searches may have missed guidelines and been biased towards English language literature.

Secondly, the guidelines were published in a range of languages. We have used native speakers where possible, but we have also had to make extensive use of translation software. This risks losing the finer nuances of a complex topic. We had to exclude Iranian guidelines from the discussion altogether because we could not secure details of their origin and methods with confidence.

Thirdly, AGREE II assumes a non-emergent process. Although the authors are confident of its applicability to a variety of settings, it was designed for guidelines produced by large teams in non-urgent conditions.

Finally, this review is limited by its cross sectional nature. We acknowledge and appreciate that more guidelines have emerged since the early pandemic and that some included in this review have been updated. This review can act as a foundation for future research to evaluate temporal changes in the quality of clinical guidelines as the covid-19 pandemic progresses.

### Comparisons with other studies

To our knowledge, this is the first rapid review of guidelines produced during a pandemic. Previous work has retrospectively examined the quality of guidelines produced in emergencies and noted serious methodological shortcomings during their production.^3^ ^4^ Our study confirms the need for a rigorous method of producing guidelines during a public health emergency of international concern.

### Conclusions and policy implications

This review has lessons for clinicians, stakeholders, and governments facing future outbreaks. Guideline development in a pandemic is extremely challenging. A flexible yet robust method of producing guidelines during an emergency is needed, recognising the contingent nature of the evidence while guaranteeing essential methodological rigour and providing a mechanism for regular review.

We suggest three components that are crucial for the production of emergency guidelines. Firstly, all guidelines produced in an emergency should be considered “living” guidelines and produced with set, transparent timelines for revision and amendment. Secondly, all guidelines produced in an emergency should use a transparent framework for weighing the strength of their recommendations (for example, GRADE or ADAPTE), so that users can understand the mechanism whereby recommendations have been made in uncertainty. Thirdly, all guidelines produced in an emergency should be externally reviewed using a validated tool such as AGREE II, to highlight areas in which they are vulnerable and to allow the authors to remedy these deficiencies in future revisions of their “living” guideline. Ensuring that WHO has the resources to provide the best possible guidelines during an emergency and for these to be updated meticulously is also vital. Our review found comprehensive guidelines already written for related respiratory infections.^37^ ^38^ ^39^ Building on established guidelines and adapting them could become a key part of any future emergency pandemic response.

The validity of currently available clinical guidelines for covid-19 will not be known for some time. This review highlights areas for improvements ahead of the next public health emergency of international concern.

**What is already known on this topic**

Clinical guidelines produced in previous healthcare emergencies have fallen below gold standards of guideline development
During the early coronavirus pandemic, a high degree of uncertainty existed about the optimal clinical management of patients with covid-19

**What this study adds**

Clinical guidelines written in the early covid-19 pandemic possessed methodological weaknesses, especially in the rigour of their development
Recommendations for the management of vulnerable groups such as older people were also neglected
Guidelines produced early in future pandemics should prioritise contingency, adaptability, and methodological rigour

## Web extra

Extra material supplied by authors

Web appendix: Supplementary material

## Notes

Contributors: AD led this project, designing the protocol, overseeing screening and data extraction, and writing the manuscript. LS helped to design the research protocol, provided advice on strategy, and participated in screening and data extraction. EH formed the search strategy and executed the database search. PB, IR, VC, SL, and EC screened the references, assisted with data extraction, and provided comments on analysis and interpretation of the data. PH is the group leader and oversaw the project, providing leadership on the research protocol and data interpretation. AD and LS had full access to all of the data, including statistical reports and tables, and take full responsibility for the integrity of the data and the accuracy of its analysis. The corresponding author attests that all listed authors meet authorship criteria and that no others meeting the criteria have been omitted. AD and LS are the guarantors.

Ethical approval: Not needed.

Data sharing: No additional data available.

The lead author affirms that this manuscript is an honest, accurate, and transparent account of the study being reported; that no important aspects of the study have been omitted; and that any discrepancies from the study as planned (and, if relevant, registered) have been explained.

Dissemination to participants and related patient and public communities: No patients were involved in this review. The research findings will be disseminated using the network of international researchers, ISARIC, which comprises over 100 international groups studying epidemic readiness. Additionally, it will be disseminated through ALERRT, a group of Africa specific research groups. The research will be displayed on the Epidemic diseases Research Group Oxford (ERGO) website and through its social media service.
