# Supplementary material for: Scope, quality, and inclusivity of clinical guidelines produced early in the covid-19 pandemic: rapid review
Source: BMJ. 2020 May 26;369:m1936. doi: 10.1136/bmj.m1936 (PMC7249097; doi:10.1136/bmj.m1936)
Supplement: Supplementary file 1 — Web appendix: Supplementary material [file daga056326.ww1.pdf]

Drew Dagens: NDM TropMed

A Systematic Review of the Availability, Quality and Inclusivity of Supportive Care Guidelines in the Management of High Consequence Infectious Disease

Search strategies run on 06/02/2020 by Eli Harriss, a librarian at the Bodleian Health Care Libraries, University of Oxford. Please acknowledge my support in any publications related to these searches, or allow me to be a coauthor on the final publication.

Note that the guidelines search filter has been adapted from the CADTH website, the reference for which is as follows:

Strings attached: CADTH database search filters [Internet]. Ottawa: CADTH; 2016. [cited 06/02/2020]. Available from: https://www.cadth.ca/resources/finding-evidence/strings-attached-cadths-database-search-filters#guide

Search Strategies

Database: Medline (Ovid MEDLINE® Epub Ahead of Print, In-Process & Other Non-Indexed Citations, Ovid MEDLINE® Daily and Ovid MEDLINE®) 1946 to present

Search Strategy:

----

1 exp clinical pathway/ (6570)

2 exp clinical protocol/ (164446)

3 exp consensus/ (12107)

4 exp consensus development conference/ (11782)

5 exp consensus development conferences as topic/ (2796)

6 critical pathways/ (6570)

7 exp guideline/ (33310)

8 guidelines as topic/ (39132)

9 exp practice guideline/ (26398)

10 practice guidelines as topic/ (115007)

11 (guideline or practice guideline or consensus development conference or consensus development conference, NIH).pt. (42546)

12 (standards or guideline or guidelines).ti,kf,kw. (106765)

13 ((practice or treatment* or clinical) adj guideline*).ab. (38495)

14 (position statement* or policy statement* or practice parameter* or best practice*).ti,ab,kf,kw. (31655)

15 (CPG or CPGs).ti. (5610)

16 consensus*.ti,kf,kw. (25114)

17 ((critical or clinical or practice) adj2 (path or paths or pathway or pathways or protocol*)).ti,ab,kf,kw. (19484)

18 recommendat*.ti,kf,kw. (39563)
19 (care adj2 (standard or path or paths or pathway or pathways or map or maps or plan or plans)).ti,ab,kf,kw. (56205)
20 (algorithm* adj2 (screening or examination or test or tested or testing or assessment* or diagnosis or diagnoses or diagnosed or diagnosing or pharmacotherap* or therap* or treatment* or intervention*)).ti,ab,kf,kw. (16631)
21 1 or 2 or 3 or 4 or 5 or 6 or 7 or 8 or 9 or 10 or 11 or 12 or 13 or 14 or 15 or 16 or 17 or 18 or 19 or 20 (583075)
22 exp Hemorrhagic Fevers, Viral/ (27068)
23 ebolavirus/ or marburgvirus/ (3417)
24 Lassa Fever/ (623)
25 Hemorrhagic Fever Virus, Crimean-Congo/ or Hemorrhagic Fever, Crimean/ (1157)
26 Rift Valley Fever/ (1129)
27 (ebola* or ebov or marburg* or lassa* or CCHF or "crimean-congo*" or "congo virus" or (crimean adj2 (hemorrhagic or haemorrhagic)) or "rift valley*" or RVF).ti,ab. (15343)
28 22 or 23 or 24 or 25 or 26 or 27 (34769)
29 coronavirus infections/ or severe acute respiratory syndrome/ (8428)
30 ("middle east* respiratory syndr*" or MERS-CoV or "novel CoV*" or "novel betacoronavirus" or "novel coronavirus*" or ("middle east" adj3 (coronavirus* or cov or betacoronavirus*)) or (MERS adj3 (coronavirus* or cov or betacoronavirus*)) or "mers-coronavirus" or "mers cov" or merscov).tw. (2593)
31 ("wuhan flu" or 2019-nCoV).tw. (53)
32 (h1n1 or h5n1).tw. (22049)
33 "zoonotic influenza*".tw. (117)
34 Influenza A Virus, H1N1 Subtype/ (14999)
35 Influenza A Virus, H5N1 Subtype/ (5950)
36 29 or 30 or 31 or 32 or 33 or 34 or 35 (36109)
37 exp Henipavirus/ (679)
38 Henipavirus Infections/ (466)
39 (nipah or hendra).tw. (1147)
40 Monkeypox virus/ or Monkeypox/ (458)
41 (monkeypox or "monkey pox").tw. (750)
42 Chikungunya virus/ or Chikungunya Fever/ (3186)
43 Chikungunya.tw. (4909)
44 "Severe Fever with Thrombocyto* Syndrome".tw. (521)
45 SFTS.tw. (833)
46 Plague/ (5127)
47 plague.tw. (8942)
48 "black death".tw. (246)
49 "pathogen x".tw. (47)
50 37 or 38 or 39 or 40 or 41 or 42 or 43 or 44 or 45 or 46 or 47 or 48 or 49 (18511)

51 21 and 28 (573)

52 21 and 36 (653)

53 21 and 50 (157)

Database: Embase 1974 to present

Search Strategy:

----

1 exp clinical pathway/ (8341)

2 exp clinical protocol/ (97695)

3 exp consensus/ (67513)

4 exp consensus development conference/ (24177)

5 exp consensus development conferences as topic/ (24177)

6 critical pathways/ (8341)

7 exp practice guideline/ (535547)

8 guidelines as topic/ (366943)

9 exp practice guideline/ (535547)

10 practice guidelines as topic/ (300656)

11 (standards or guideline or guidelines).ti,kw. (148899)

12 ((practice or treatment* or clinical) adj guideline*).ab. (58666)

13 (position statement* or policy statement* or practice parameter* or best practice*).ti,ab,kw. (46408)

14 (CPG or CPGs).ti. (6767)

15 consensus*.ti,kw. (31714)

16 ((critical or clinical or practice) adj2 (path or paths or pathway or pathways or protocol*)).ti,ab,kw. (30587)

17 recommendat*.ti,kw. (50325)

18 (care adj2 (standard or path or paths or pathway or pathways or map or maps or plan or plans)).ti,ab,kw. (99334)

19 (algorithm* adj2 (screening or examination or test or tested or testing or assessment* or diagnosis or diagnoses or diagnosed or diagnosing or pharmacotherap* or therap* or treatment* or intervention*)).ti,ab,kw. (24363)

20 1 or 2 or 3 or 4 or 5 or 6 or 7 or 8 or 9 or 10 or 11 or 12 or 13 or 14 or 15 or 16 or 17 or 18 or 19 (888295)

21 virus hemorrhagic fever/ (3141)

22 Ebola hemorrhagic fever/ or Marburg hemorrhagic fever/ (5702)

23 lassa fever/ or lassa virus/ (1613)

24 Crimean Congo hemorrhagic fever/ (1134)

25 Rift Valley fever/ (877)

26 (ebola* or ebov or marburg* or lassa* or CCHF or "crimean-congo*" or "congo virus" or (crimean adj2 (hemorrhagic or haemorrhagic)) or "rift valley*" or RVF).ti,ab. (18386)

27 21 or 22 or 23 or 24 or 25 or 26 (21968)

28 exp coronavirinae/ or coronavirus infection/ or severe acute respiratory syndrome/ (16337)

29 ("middle east* respiratory syndr*" or MERS-CoV or "novel CoV*" or "novel betacoronavirus" or "novel coronavirus*" or ("middle east" adj3 (coronavirus* or cov or betacoronavirus*)) or (MERS adj3 (coronavirus* or cov or betacoronavirus*)) or "mers-coronavirus" or "mers cov" or merscov).tw. (2932)

30 ("wuhan flu" or 2019-nCoV).tw. (32)

31 (h1n1 or h5n1).tw. (27461)

32 "zoonotic influenza*".tw. (130)

33 exp "influenza a virus (h1n1)"/ (3792)

34 "influenza a virus (h5n1)"/ (1281)

35 28 or 29 or 30 or 31 or 32 or 33 or 34 (45354)

36 exp henipavirus/ (1283)

37 exp Henipavirus infection/ (502)

38 (nipah or hendra).tw. (1318)

39 monkeypox/ or monkeypox virus/ (786)

40 (monkeypox or "monkey pox").tw. (830)

41 Chikungunya virus/ or chikungunya/ (4882)

42 Chikungunya.tw. (6173)

43 "Severe Fever with Thrombocyto* Syndrome".tw. (565)

44 SFTS.tw. (985)

45 plague/ (5904)

46 plague.tw. (7797)

47 "black death".tw. (253)

48 "pathogen x".tw. (41)

49 36 or 37 or 38 or 39 or 40 or 41 or 42 or 43 or 44 or 45 or 46 or 47 or 48 (20982)

50 20 and 27 (789)

51 20 and 35 (1284)

52 20 and 49 (382)

Database: Global Health <1973 to 2020 Week 05>

Search Strategy:

----

1 exp consensus/ (38)

2 guidelines/ (42871)

3 (standards or guideline or guidelines).ti. (11939)

4 ((practice or treatment* or clinical) adj guideline*).ab. (5437)

5 (position statement* or policy statement* or practice parameter* or best practice*).ti,ab. (4691)

6 (CPG or CPGs).ti. (481)

7 consensus*.ti. (1891)

8 ((critical or clinical or practice) adj2 (path or paths or pathway or pathways or protocol*)).ti,ab. (1485)

9 recommendat*.ti. (6283)

10 (care adj2 (standard or path or paths or pathway or pathways or map or maps or plan or plans)).ti,ab. (5259)

11 (algorithm* adj2 (screening or examination or test or tested or testing or assessment* or diagnosis or diagnoses or diagnosed or diagnosing or pharmacotherap* or therap* or treatment* or intervention*)).ti,ab. (1743)

12 1 or 2 or 3 or 4 or 5 or 6 or 7 or 8 or 9 or 10 or 11 (65662)

13 haemorrhagic fevers/ (5098)

14 exp Ebola haemorrhagic fever/ (558)

15 exp marburgvirus/ (712)

16 exp lassa virus/ (792)

17 exp crimean-congo haemorrhagic fever virus/ (1513)

18 exp rift valley fever virus/ (1695)

19 (ebola* or ebov or marburg* or lassa* or CCHF or "crimean-congo*" or "congo virus" or (crimean adj2 (hemorrhagic or haemorrhagic)) or "rift valley*" or RVF).ti,ab. (8739)

20 13 or 14 or 15 or 16 or 17 or 18 or 19 (12292)

21 exp betacoronavirus/ (3784)

22 ("middle east* respiratory syndr*" or MERS-CoV or "novel CoV*" or "novel betacoronavirus" or "novel coronavirus*" or ("middle east" adj3 (coronavirus* or cov or betacoronavirus*)) or (MERS adj3 (coronavirus* or cov or betacoronavirus*)) or "mers-coronavirus" or "mers cov" or merscov).tw. (1317)

23 ("wuhan flu" or 2019-nCoV).tw. (0)

24 (h1n1 or h5n1).tw. (13124)

25 "zoonotic influenza*".tw. (112)

26 21 or 22 or 23 or 24 or 25 (16851)

27 exp henipavirus/ (995)

28 (nipah or hendra).tw. (998)

29 exp monkeypox virus/ (358)

30 (monkeypox or "monkey pox").tw. (454)

31 exp chikungunya virus/ (3643)

32 Chikungunya.tw. (4232)

33 "Severe Fever with Thrombocyto* Syndrome".tw. (427)

34 SFTS.tw. (399)

35 plague/ (3069)

36 plague.tw. (7171)

37 "black death".tw. (93)

38 "pathogen x".tw. (3)

39 27 or 28 or 29 or 30 or 31 or 32 or 33 or 34 or 35 or 36 or 37 or 38 (13219)

40 12 and 20 (279)

41 12 and 26 (463)

42 12 and 39 (157)

Scopus

Cluster 1

((TITLE-ABS-KEY ("clinical pathway*" OR "clinical protocol*" OR consensus OR guideline* OR "position statement*" OR "policy statement*" OR "practice parameter*" OR "best practice*") OR TITLE-ABS-KEY ((care W/2 (standard OR path OR paths OR pathway OR pathways OR map OR maps OR plan OR plans))) OR TITLE-ABS-KEY ((algorithm* W/2 (screening OR examination OR test OR tested OR testing OR assessment* OR diagnosis OR diagnoses OR diagnosed OR diagnosing))) OR TITLE-ABS-KEY ((algorithm* W/2 (pharmacotherap* OR therap* OR treatment* OR intervention*))) OR TITLE (standards OR recommendat*))) AND ((TITLE-ABS-KEY (((virus OR viral) W/3 ("hemorrhagic fever*" OR "haemorrhagic fever*")) OR ebola* OR ebov* OR marburg* OR lassa) OR TITLE-ABS-KEY (cchf OR "crimean-congo*" OR "congo virus" OR (crimean W/2 (hemorrhagic OR haemorrhagic)) OR "rift valley" OR rvf)))

Cluster 2

((TITLE-ABS-KEY ("clinical pathway*" OR "clinical protocol*" OR consensus OR guideline* OR "position statement*" OR "policy statement*" OR "practice parameter*" OR "best practice*") OR TITLE-ABS-KEY ((care W/2 (standard OR path OR paths OR pathway OR pathways OR map OR maps OR plan OR plans))) OR TITLE-ABS-KEY ((algorithm* W/2 (screening OR examination OR test OR tested OR testing OR assessment* OR diagnosis OR diagnoses OR diagnosed OR diagnosing))) OR TITLE-ABS-KEY ((algorithm* W/2 (pharmacotherap* OR therap* OR treatment* OR intervention*))) OR TITLE (standards OR recommendat*))) AND ((TITLE-ABS-KEY ("middle east* respiratory syndr*" OR mers-cov OR "novel CoV*" OR "novel betacoronavirus" OR "novel coronavirus*") OR TITLE-ABS-KEY (("middle east" W/3 (coronavirus* OR cov OR betacoronavirus*)) OR (mers W/3 (coronavirus* OR cov OR betacoronavirus*)) OR "mers-coronavirus" OR "mers cov" OR merscov) OR TITLE-ABS-KEY (("wuhan flu" OR 2019-ncov)) OR TITLE-ABS-KEY (h1n1 OR h5n1) OR TITLE-ABS-KEY ("zoonotic influenza*")))

Cluster 3

((TITLE-ABS-KEY ("clinical pathway*" OR "clinical protocol*" OR consensus OR guideline* OR "position statement*" OR "policy statement*" OR "practice parameter*" OR "best practice*") OR TITLE-ABS-KEY ((care W/2 (standard OR path OR paths OR pathway OR pathways OR map OR maps OR plan OR plans))) OR TITLE-ABS-KEY ((algorithm* W/2 (screening OR examination OR test OR tested OR testing OR assessment* OR diagnosis OR diagnoses OR diagnosed OR diagnosing))) OR TITLE-ABS-KEY ((algorithm* W/2 (pharmacotherap* OR therap* OR treatment* OR intervention*))) OR TITLE (standards OR recommendat*))) AND (TITLE-ABS-KEY (henipavirus OR nipah OR hendra OR monkeypox OR "monkey pox" OR chikungunya OR "Severe Fever with Thrombocyto* Syndrome" OR sfts OR plague OR "black death" OR "pathogen x"))

Web of Science Core Collection

#1 TOPIC: ("clinical pathway*" OR "clinical protocol*" OR consensus OR guideline* OR "position statement*" OR "policy statement*" OR "practice parameter*" OR "best practice*" OR CPG OR CPGs) OR TOPIC: ((care near/2 (standard or path or paths or pathway or pathways or map or maps or plan or plans))) OR TOPIC: ((algorithm* near/2 (screening or

examination or test or tested or testing or assessment* or diagnosis or diagnoses or diagnosed or diagnosing))) OR TOPIC: ((algorithm* near/2 (pharmacotherap* or therap* or treatment* or intervention*))) OR TITLE: (standards OR recommendat*)
#2 TOPIC: (((virus or viral) near/3 ("hemorrhagic fever*" OR "haemorrhagic fever*")) OR ebola* OR ebov* OR marburg* OR lassa OR CCHF or "crimean-congo*" or "congo virus" or (crimean near/2 (hemorrhagic or haemorrhagic)) OR "rift valley" OR RVF)
#3 TOPIC: ("middle east* respiratory syndr*" or MERS-CoV or "novel CoV*" or "novel betacoronavirus" or "novel coronavirus*") OR TOPIC: (("middle east" near/3 (coronavirus* or cov or betacoronavirus*)) or (MERS near/3 (coronavirus* or cov or betacoronavirus*)) or "mers-coronavirus" or "mers cov" or merscov) OR TOPIC: (("wuhan flu" or 2019-nCoV)) OR TOPIC: (h1n1 or h5n1) OR TOPIC: ("zoonotic influenza*")
#4 TOPIC: (Henipavirus OR nipah or hendra or monkeypox or "monkey pox" OR Chikungunya OR "Severe Fever with Thrombocyto* Syndrome" OR SFTS OR plague OR "black death" OR "pathogen x")
#5 #2 AND #1
#6 #3 AND #1
#7 #4 AND #1

The WHO Global Index Medicus Regional Libraries
https://pesquisa.bvsalud.org/gim/?lang=en
Cluster 1
(tw:("clinical path*" OR "clinical protocol*" or "critical path*" OR "critical protocol*" OR "practice path*" OR "practice protocol*" OR consensus OR guideline* OR standards OR "position statement*" OR "policy statement*" OR "practice parameter*" OR "best practice*" OR CPG OR CPGs OR "care standard" OR "care path*" OR "care map*" OR "care plan*" OR algorithm*)) AND (tw:(("viral hemorrhagic fever*" or "hemorrhagic fever* virus*" OR "viral haemorrhagic fever*" or "haemorrhagic fever* virus*" OR ebola* OR ebov* OR marburg* OR lassa OR CCHF or "crimean-congo*" or "congo virus" or "crimean hemorrhagic" or "crimean haemorrhagic" OR "rift valley" OR RVF)))
Cluster 2
(tw:("clinical path*" OR "clinical protocol*" or "critical path*" OR "critical protocol*" OR "practice path*" OR "practice protocol*" OR consensus OR guideline* OR standards OR "position statement*" OR "policy statement*" OR "practice parameter*" OR "best practice*" OR CPG OR CPGs OR "care standard" OR "care path*" OR "care map*" OR "care plan*" OR algorithm*)) AND (tw:(("middle east* respiratory syndr*" or MERS-CoV or "novel CoV*" or "novel betacoronavirus" or "novel coronavirus*") or ("middle east" and (coronavirus* or cov or betacoronavirus*)) or (MERS near/3 (coronavirus* or cov or betacoronavirus*)) or "mers-coronavirus" or "mers cov" or merscov or ("wuhan flu" or 2019-nCoV) or h1n1 or h5n1 or "zoonotic influenza*"))
Cluster 3
(tw:("clinical path*" OR "clinical protocol*" or "critical path*" OR "critical protocol*" OR "practice path*" OR "practice protocol*" OR consensus OR guideline* OR standards OR "position statement*" OR "policy statement*" OR "practice parameter*" OR "best practice*" OR CPG OR CPGs OR "care standard" OR "care path*" OR "care map*" OR "care plan*" OR algorithm*)) AND (tw:(Henipavirus OR nipah or hendra or monkeypox or "monkey pox" OR Chikungunya OR "Severe Fever with Thrombocyto* Syndrome" OR SFTS OR plague OR "black death" OR "pathogen x"))

Search Results

**Database**	**Cluster 1 results**	**Cluster 2 results**	**Cluster 3 results**
Ovid Medline	573	653	157
Ovid Embase	789	1284	382
Ovid Global Health	279	463	157
Scopus	1106	1742	725
Web of Science Core Collection	609	928	722
WHO Global Index Medicus	25	148	85
TOTAL	3381	5218	2228
Total after deduplication	1761	2996	1428

Google Scholar – Screen the first 10 pages of results
Sorted by relevance:

Cluster 1
(guideline|consensus|standards|"clinical path*"|"clinical protocol*"|"practice path*"|"position statement*"|"policy statement*"|"best practice*")("viral hemorrhagic fever"|ebola*|ebov*|lassa|Marburg|CCHF|Crimean-congo|"congo virus*"|"rift valley")
https://scholar.google.co.uk/scholar?hl=en&as_sdt=0%2C5&q=%28guideline%7Cconsensus%7Cstandards%7C%22clinical+path*%E2%80%9D%7C%22clinical+protocol*%22%7C%22practice+path*%22%7C%22position+statement*%22%7C%22policy+statement*%22%7C%22best+practice*%22%29%28%22viral+hemorrhagic+fever%22%7Cebola*%7Cebov*%7Classa%7CMarburg%7CCCHF%7CCrimean-congo%7C%22congo+virus*%22%7C%22rift+valley%22%29&btnG=

Cluster 2
(guideline|consensus|standards|"clinical path*"|"clinical protocol*"|"practice path*"|"position statement*"|"policy statement*"|"best practice*")("middle east resp* syndrome*"|mers*|"novel coronavirus*"|"middle east coronavirus*"|2019-nCoV|h1n1|h5n1|"zoonotic influenza*")
https://scholar.google.co.uk/scholar?hl=en&as_sdt=0%2C5&q=%28guideline%7Cconsensus%7Cstandards%7C%22clinical+path*%E2%80%9D%7C%22clinical+protocol*%22%7C%22practice+path*%22%7C%22position+statement*%22%7C%22policy+statement*%22%7C

%22best+practice*%22%29%28%22middle+east+resp*+syndrome*%22%7Cmers*%7C%22novel+coronavirus*%22%7C%22middle+east+coronavirus*%22%7C2019-nCoV%7Ch1n1%7Ch5n1%7C%22zoonotic+influenza*%22%29&btnG=

Cluster 3
(guideline|consensus|standards|"clinical path*”|"clinical protocol*"|"practice path*"|" policy statement*"|"best practice*")(henipavirus|nipah|hendra|monkeypox|chickungunya|"severe fever with thrombocyte* syndrome"|sfts|"black death"|plague|"pathogen x")
https://scholar.google.co.uk/scholar?hl=en&as_sdt=0%2C5&q=%28guideline%7Cconsensus%7Cstandards%7C%22clinical+path*%E2%80%9D%7C%22clinical+protocol*%22%7C%22practice+path*%22%7C%22+policy+statement*%22%7C%22best+practice*%22%29%28henipavirus%7Cnipah%7Chendra%7Cmonkeypox%7Cchickungunya%7C%22severe+fever+with+thrombocyte*+syndrome%22%7Csfts%7C%22black+death%22%7Cplague%7C%22pathogen+x%22%29&btnG=
